# Systematic evaluation of combined herbal adjuvant therapy for proliferative diabetic retinopathy

**DOI:** 10.3389/fendo.2023.1157189

**Published:** 2023-05-18

**Authors:** Baogeng Huai, Baosha Huai, Zhenghua Su, Min Song, Changling Li, Yingjuan Cao, Tao Xin, Deshan Liu

**Affiliations:** ^1^ First Clinical Medical College, Shandong University of Traditional Chinese Medicine, Jinan, China; ^2^ Department of Traditional Chinese Medicine, Qilu Hospital, Cheeloo College of Medicine, Shandong University, Jinan, China; ^3^ Department of Ophthalmology, Qilu Hospital, Cheeloo College of Medicine, Shandong University, Jinan, China; ^4^ Department of Nursing, Qilu Hospital, Cheeloo College of Medicine, Shandong University, Jinan, China; ^5^ Department of Neurosurgery, The First Affiliated Hospital of Shandong First Medical University & Shandong Provincial Qianfoshan Hospital, Jinan, China

**Keywords:** traditional Chinese medicine, proliferative diabetic retinopathy, meta analysis, diabetic complications, Chinese medicinal herb

## Abstract

**Objective:**

To evaluate the efficacy and safety of combined traditional Chinese medicine in the adjuvant treatment of proliferative diabetic retinopathy (PDR) by Meta-analysis.

**Methods:**

PubMed, Embase, Web of Science, Cochrane Library, China National Knowledge Infrastructure (CNKI), Wanfang databases were searched by computer. Random controlled clinical trials (RCTS) using traditional Chinese medicine as adjuvant therapy for proliferative diabetic retinopathy were screened, and Stata16.0 software was used to perform meta-analysis on the final included literatures.

**Results:**

A total of 18 studies involving 1392 patients were included. Meta-analysis showed that the clinical effective rate OR=2.99 (*CI*: 2.18-4.10, *I^2^ = *42.7%, *P*<0.05); Visual acuity MD=0.10(*CI*: 0.06-0.13, *I^2^ = *0%, *P*<0.05); Fundus efficacy OR=5.47 (*CI*: 1.33-22.51, *I^2^ = *71.4%, *P*<0.05); Neovascularisation regression rate OR=8 (*CI*: 3.83-16.71, *I^2^ = *30.1%, *P*<0.05); Macular foveal thickness MD=-44.24 (*CI*: -84.55–3.93, *I^2^ = *95.6%, *P*<0.05); Absorption of vitreous hemorrhage OR=4.7 (*CI*: 2.26-9.77, *I^2^ = *0%, *P*<0.05); Fasting blood glucose MD=-0.23, (*CI*: -0.38–0.07, *I^2^ = *0%, *P*<0.05); 2h postprandial blood glucose MD=-0.19 (*CI*: -0.52-0.14, *I^2^ = *0%, *P*=0.25). From the results, the combined Chinese medicine adjuvant therapy showed better efficacy than the control group. A total of 69 kinds of traditional Chinese medicine were involved in 18 studies, among which the top four applied frequencies were Panax notoginseng, Rehmannia rehmannii, Astragalus membranaceus and Poria cocos. Most of the medicines were sweet and bitter in taste, the qi tended to be slight cold and cold, and the meridian tropism belongs to the liver meridian.

**Conclusion:**

The combination of traditional Chinese medicine adjuvant therapy has a good curative effect on PDR patients. However, the relevant clinical trials are few and more high-quality clinical trials are still needed, what’s more the attention should be paid to the exploration of its safety.

## 1 Introduction

Diabetic retinopathy (DR) is one of the most common clinical complications of diabetes mellitus (DM). Of the 537 million diabetic patients worldwide, DR accounts for about 22.27% and has become the leading cause of blindness in middle-aged and older adults (1). As a disease with a large potential patient population base and a high probability of blindness and disability, DR brings serious life hindrances and mental suffering to patients while adding heavy economic pressure and burden to society. As the world’s largest diabetes population, China has a staggering 140.9 million people with diabetes, with the number of diabetic retinopathy patients estimated to be between 34 and 53 million (2). The search for clinical drugs that can effectively treat diabetic retinopathy is an urgent problem worldwide, especially in China.

Chinese medicine has been handed down in China for thousands of years. A large amount of research evidence shows that it has excellent therapeutic effects on diabetes and its complications by reducing blood glucose, lipid profile and improving other metabolic indicators (3). At the same time, many studies have also proved that Chinese herbal therapy has a better intervention effect on DR (4, 5). In China’s extensive clinical practice, not only traditional Chinese medicine practitioners commonly utilize herbal remedies to treat DR, while Western medical practitioners also frequently incorporate certain Chinese patent medicines to complement their treatment regimens for DR.

However, it is worth noting that DR can be divided into two types: Non-proliferative diabetic retinopathy (NPDR) and Proliferative diabetic retinopathy (PDR) based on the presence of neovascularization, and the two have distinct pathological features (6). Currently, research on TCM treatment for DR in China focuses mostly on the NPDR stage, where clinical manifestations include blood stasis lesions such as retinal microaneurysms and venous beading, and herbal remedies are often used to improve retinal microcirculation, with their efficacy widely recognized (7). However, the effectiveness and safety of TCM treatment for PDR, in which new blood vessels grow in the retina, are still debated. High-level research evidence is urgently needed to determine whether TCM treatment can help improve clinical symptoms in PDR patients and whether it is safe to use in this stage. To this end, we conducted this study with the aim of addressing this controversy. Additionally, to better guide the selection and subsequent research of TCM treatments, we summarized the frequency, four natures and five flavors, and meridian tropism of the herbal medicines included in the studies, aiming to explore the laws of TCM treatment for PDR.

## 2 Methods

### 2.1 Literature search

This study searched PubMed, Web of Science, Cochrane Library, Embase database, China Knowledge Network, Wanfang to include randomized clinical controlled trials of combination treatment of PDR with Chinese herbs since the establishment of the database until October 8, 2022.

### 2.2 Inclusion criteria

(1) Study type: a randomized controlled trial of Chinese herbal medicine for treating PDR, language not limited.
(2) Study subjects: all included cases must meet the pathological diagnostic criteria for PDR.
(3) Interventions: The interventions in the control group can be conventional Western medical treatment, such as drugs, photocoagulation or surgery, or blank; the interventions in the trial group should be based on conventional Western medical treatment combined with the use of Chinese herbal medicine as an adjunct, which can be herbal medicine, compound prescriptions, Chinese patent medicines, injections, ion introduction.
(4) Outcome indicators: Assess post-treatment outcomes involving at least one clinical efficacy, visual acuity, blood glucose, fundus efficacy, regression of neovascularisation, macular central recess thickness, and absorption of vitreous blood accumulation.

### 2.3 Exclusion criteria

(1) Type of literature is a review, animal test, or another non-clinical trial article.
(2) Non-simultaneous randomized controlled clinical trials, lack of randomization of groupings.
(3) The treatment method does not include herbal medicine as an adjunctive treatment.
(4) Lack of observation on relevant indicators.
(5) Inappropriate statistical methods or severe errors in the data.

### 2.4 Data extraction

Two researchers (Baogeng Huai and Baosha Huai) independently screened the literature according to the above criteria using EndNote X9 software, cross-checked, and controversial literature was decided by a third party (Changling Li) in consultation. Judgments were made based on whether the study population belonged to PDR patients, whether the intervention method involved TCM treatment, and whether there were any logical loopholes or data errors in the research. An Excel sheet was used to prepare an information extraction form based on the study content, and the data were extracted as follows.

(1) Basic information about the literature: first author, date of publication.
(2) Trial subjects: sample size of the trial and control groups.
(3) Interventions: type, dosage form, a dose of Chinese herbal medicine and control drugs, and duration of intervention.
(4) Outcome indicators: clinical efficacy, visual acuity, fundus efficacy criteria, neovascularization regression rate, central macular recess thickness.

### 2.5 Evaluation of literature quality and risk of bias

The risk of bias assessment tool of the Cochrane Systematic Reviews was used to evaluate the included studies, including randomization, allocation concealment, whether blinding was used, completeness of outcome indicators, whether study results were reported selectively, and other biases. The evaluation results were categorized into three risk levels: “high risk,” “low risk,” and “uncertain.”

### 2.6 Statistical methods

The study was analyzed using Stata16.0 software. The mean difference (MD) was selected for the analysis of the effect of measures, and the odds ratio (OR) was selected as the effect size for dichotomous variables, both using a 95% confidence interval (CI). *P*-values and *I^2^* were calculated to determine whether heterogeneity existed among the included studies, and if heterogeneity was observed (*P*<0.05, or *I^2^* >50%), a random-effects model was selected; if no heterogeneity was observed (*P*>0.05, and *I^2^* ≤50%), a fixed-effects model was selected. *α* = 0.05 was used as the meta-analysis test level. Publication bias was evaluated using the funnel plot, Begg’s test, and Egger’s test.

## 3 Results

### 3.1 Basic process for inclusion in the literature

**Figure 1:**
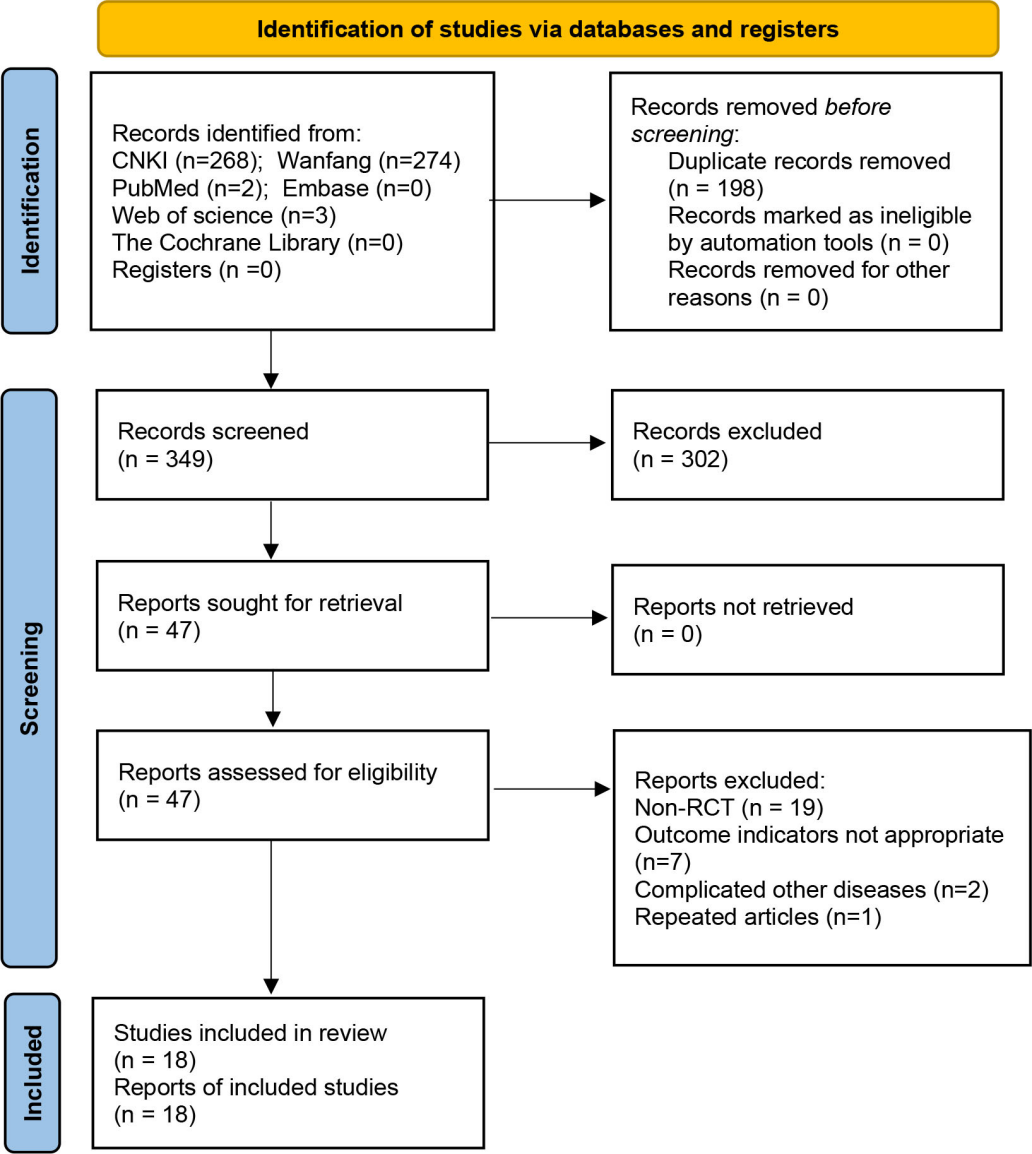
Flow chart of literature retrieval.

As of October 2022, a total of 547 studies in the literature that could be potentially relevant were retrieved. After title and abstract screening, 47 articles were reviewed in total, and 18 RCT studies (8–25) were finally included for Meta-analysis based on the inclusion criteria (**Figure 1**). There are 1392 patients altogether in the sample size of the 18 studies. The intervention group and the control group had equivalent pre-treatment data (**Table 1**).

**Table 1:** Basic information about the included literature.

Study	Average Age		Treatment Group		Control Group		Intervention		Treatment Duration	Outcomes
	Treatment Group	Control Group	Male	Female	Male	Female	Treatment Group	Control Group		
Lv 2017	51.2 ± 12.3	50.9 ± 11.4	–	–	–	–	DaMing Decoction	Calcium Dobesilate	12 W	3
Fan 2016	64.3 ± 10.3	65.4 ± 11.2	16	14	15	15	Compound XueShuanTong Capsule	Diabetic Basal Treatment	12W	3, 5
Sun 2016	–	–	–	–	–	–	HuaYuMingMu Decoction	Calcium Dobesilate	24 W	1, 2
Li 2016	41.55 ± 6.51	40.57 ± 6.48	22	21	20	22	Photocoagulation+ Lycium Rehmannia Pills	Photocoagulation	12 W	1, 2, 7
Ji 2010	–	–	–	–	–	–	TangWangZengShi Decoction	Basal Treatment	24 W	1
Niu 2010	55.97 ± 6.35	56.82 ± 7.56	8	8	9	7	DaMing Decoction	Calcium Dobesilate	4 W	1,2,3
Han 2019	54.57 ± 11.47	51.13 ± 11.84	8	9	9	6	SanQi Powder	Basal Treatment	2 W	6
Yuan 2012	–	–	–	–	–	–	Photocoagulation+Discriminated Chinese Medicne	Photocoagulation	8 W	1
Teng 2012	–	–	–	–	–	–	Argon Laser+ DaMing Decoction	Argon Laser	8 W	1, 3
Di 2007	58.6	61.2	18	32	16	15	Photocoagulation+ZiYinLiangXueSanYu Decoction	Photocoagulation	48 W	4
Wang 2012	55.47 ± 8.74	56.1 ± 8.89	18	12	19	11	Photocoagulation+ Lycium Rehmannia Pills+ XueSaiTong capsule	Photocoagulation	12 W	1, 2, 7
Zhang 2019	61.5 ± 4.6	60.7 ± 4.4	27	23	28	22	Calcium Dobesilate+ Photocoagulation+ HuoXueMingMu Tablets	Calcium Dobesilate+ Photocoagulation	12~24 W	1, 4
Wang 2020	58.24 ± 4.88	59.33 ± 5.04	59	41	60	40	Discriminated Chinese Medicne	Basal Treatment	24 W	1, 4
Li 2015	58.5	60.1	10	8	9	9	Chinese Medicine Ion Introduction	Iodized Lecithin Tablets	4 W	6
Wu 2017	56.45 ± 7.28	55.89 ± 8.34	20	21	22	19	Chinese Medicine Ion Introduction	Iodized Lecithin Tablets	4 W	6
Sha 2019	47.1 ± 7.8	48.1 ± 6.9	17	18	15	20	Minimally Invasive Vitrectomy+ ZiYinHuaYuTongLuo Prescription	Minimally Invasive Vitrectomy	4 W	5
Ma 2016	–	–	–	–	–	–	Modified BuYangHuanWu Decoction	Calcium Dobesilate	24 W	1
Zhao 2021	53.7 ± 6.7	54.5 ± 7.1	27	15	25	17	Ranibizumab Combined with PPV+Five Ling Powder and Ba Zhen Decoction	Ranibizumab Combined with PPV	12 W	1, 5

Clinical Efficacy; 2. Visual Acuity Level; 3. Fundus Efficacy; 4. Neovascularisation Regression Rate; 5. Macular Central Recess Thickness; 6. Absorption of Vitreous Hemorrhage; 7. Absorption of Vitreous Hemorrhage; "-" indicates that the data was not mentioned in the included study.

### 3.2 Evaluation of the quality of the included literature

The 18 pieces of literature were divided into six items to explore the quality of the studies. All 18 papers were organized for randomized sequence generation using the randomized order principle. 11 datasets (9–11, 13–16, 19, 20, 23, 25) used either the random number table or the coin flip method, so the bias judgment was assessed as low risk. Only one study (10) used a single-blind design, and the remaining studies did not involve blinding, so the risk of bias was assessed as high. While the remaining four were not mentioned. (There was no mention of concealment of assignment order, insufficient result data, selective reporting, or other biases.)

### 3.3 Results of meta-analysis

#### 3.3.1 Clinical efficacy

**Figure 2:**
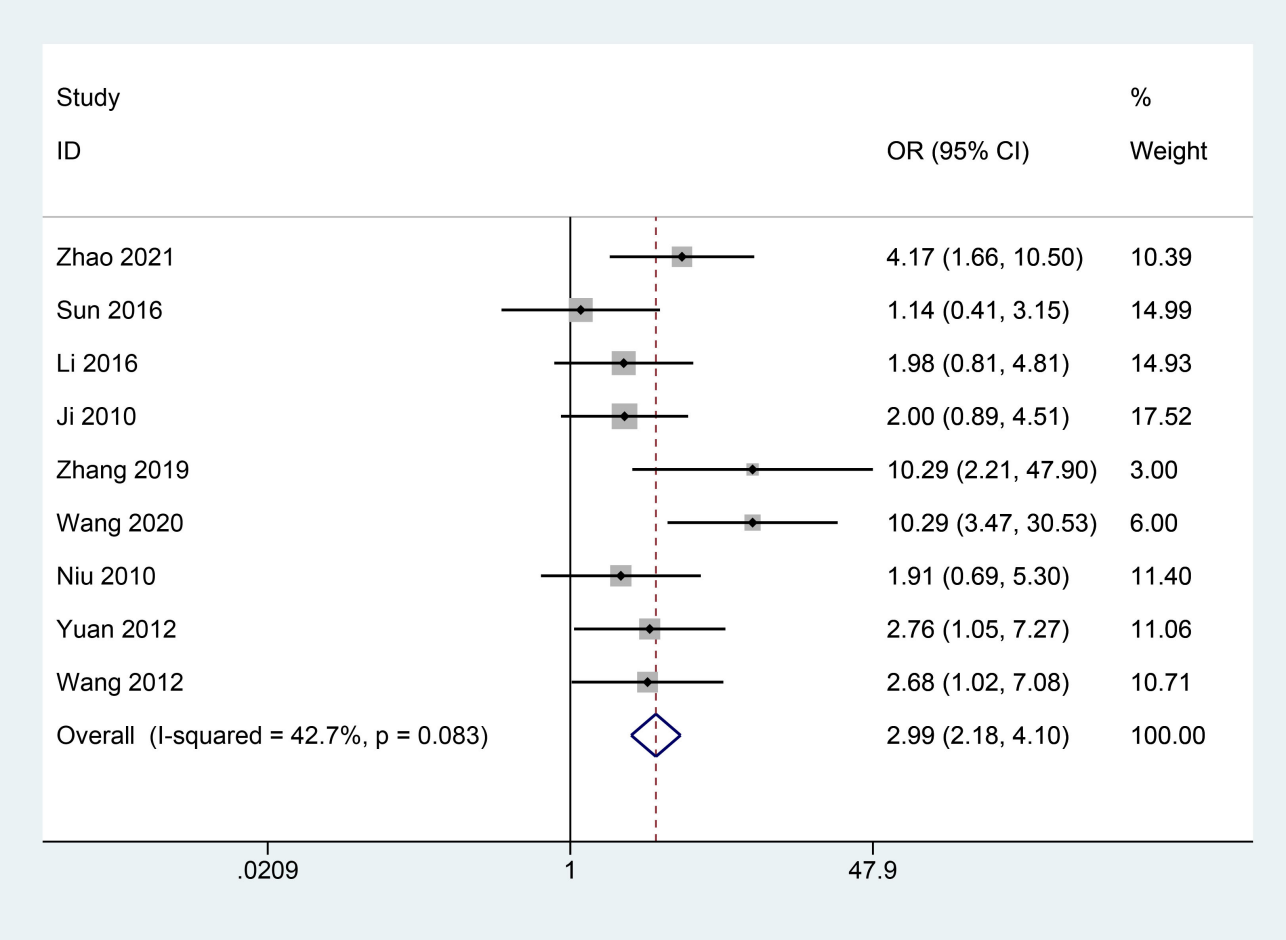
Forest plot of clinical efficacy.

A total of 9 articles (10–13, 15, 18–20, 25) reported the clinical efficacy of combined herbal treatments for patients with PDR, including 827 patients. The meta results showed that combined herbal treatments effectively improved the clinical outcomes of patients with PDR (9 studies, 827 patients, OR=2.99, *CI*: 2.18-4.10, *I^2^ = *42.7%, *P*<0.05) (**Figure 2**).

#### 3.3.2 Visual acuity level

**Figure 3:**
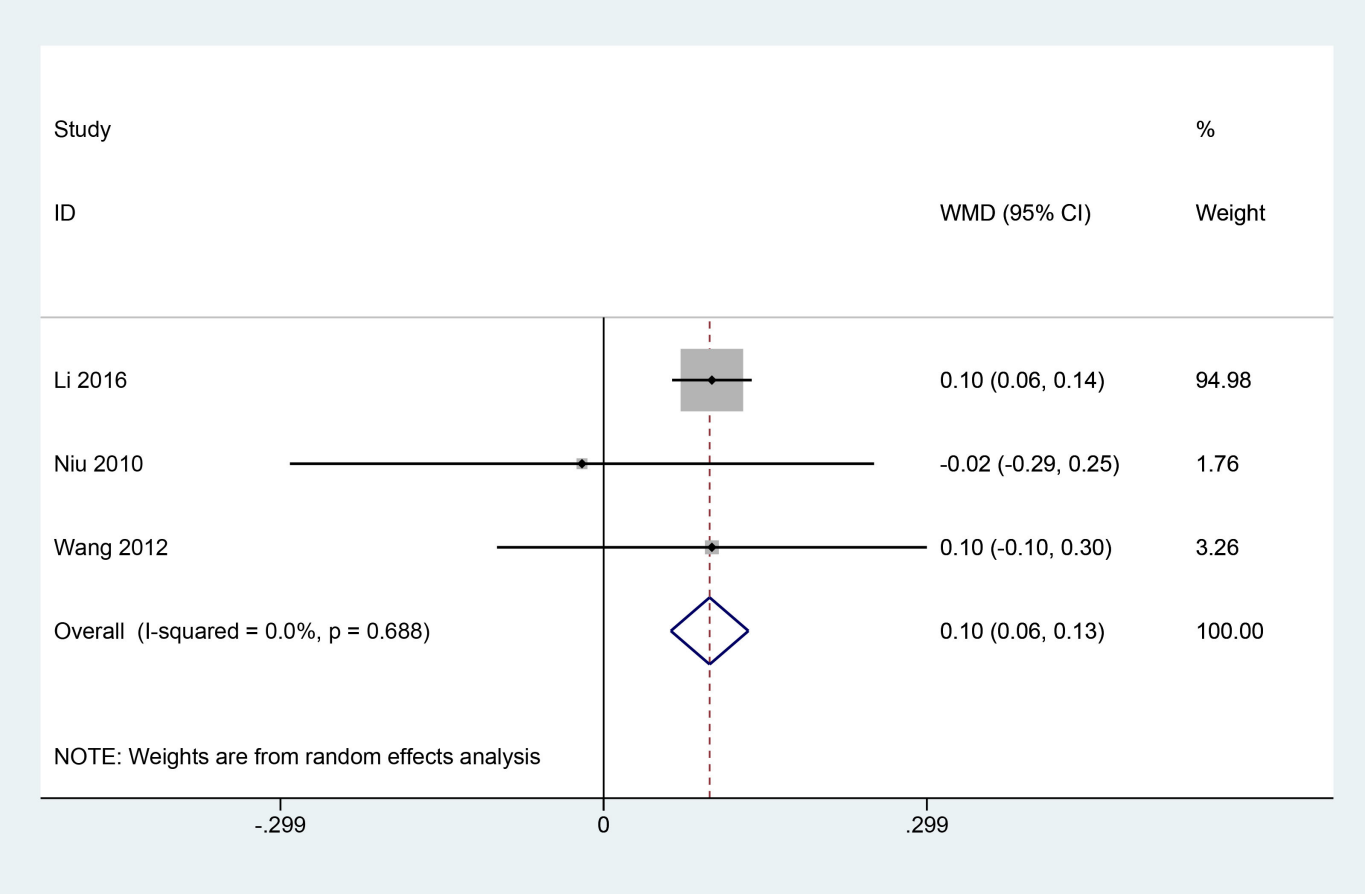
Forest plot of visual acuity level.

A total of 3 articles (11, 13, 18) reported the effect of combined herbal treatment on visual acuity in patients with PDR. 177 patients were included, and the meta results showed that combined herbal treatment could improve the visual acuity level of patients to some extent (3 studies, 177 patients, MD=0.10, *CI*: 0.06-0.13, *I^2^ = *0%, *P*<0.05) (**Figure 3**).

#### 3.3.3 Fundus efficacy

**Figure 4:**
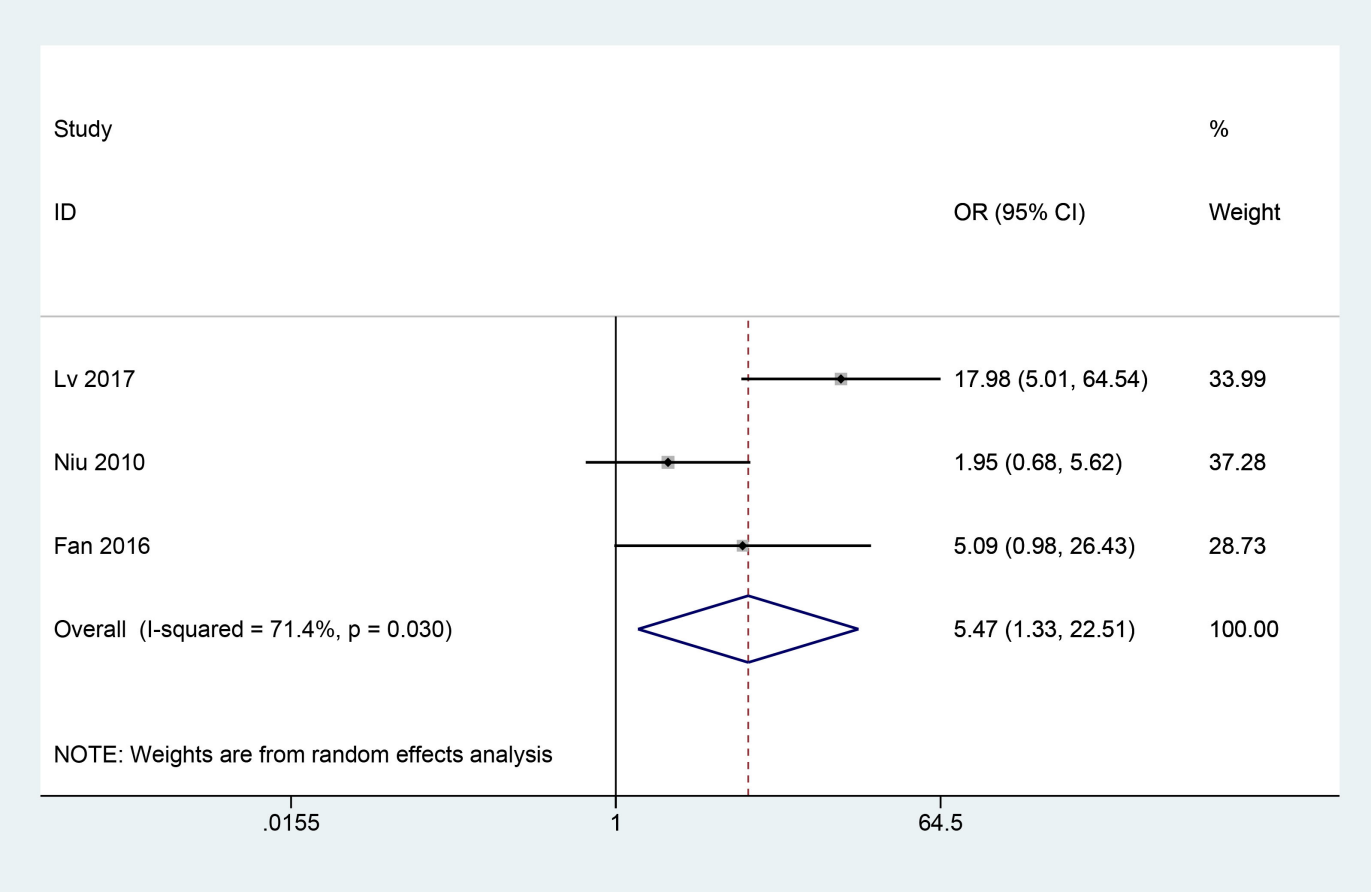
Forest plot of fundus efficacy.

A total of 3 articles (8, 9, 13) reported the fundus efficacy of combined herbal treatment in patients with PDR. 202 patients were included, and the meta results showed that combined herbal treatment significantly improved the fundus efficacy in patients with PDR (3 studies, 202 patients, OR=5.47, *CI*: 1.33-22.51, *I^2^ = *71.4%, *p*<0.05) (**Figure 4**).

#### 3.3.4 Neovascularisation regression rate

**Figure 5:**
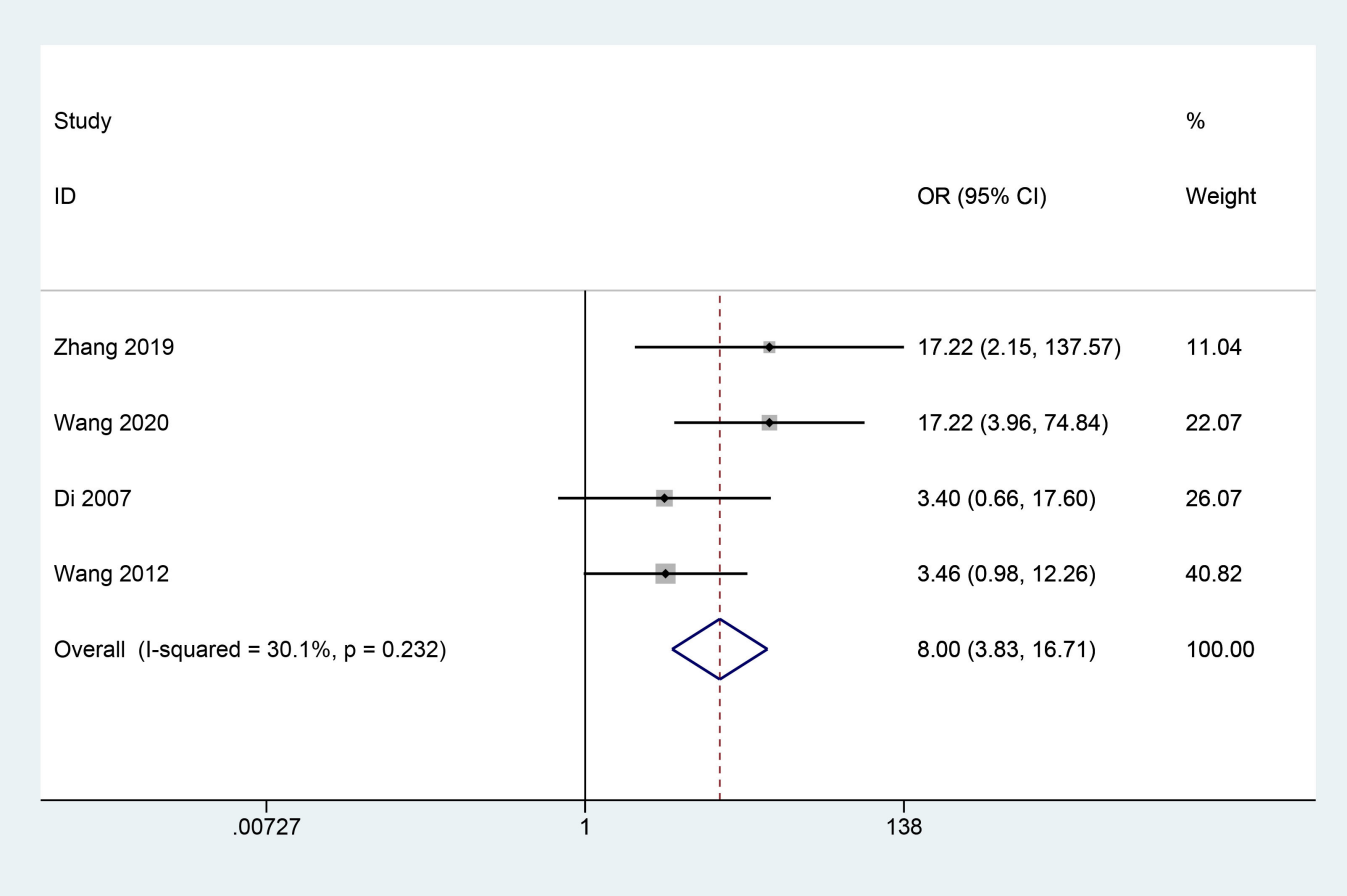
Forest plot of neovascularisation regression rate.

A total of 4 articles (17–20) reported the effect of herbal combination therapy on the regression of neovascularisation. 424 patients were included and the meta results showed that herbal combination therapy was effective in helping the regression of retinal neovascularisation in patients with PDR (4 studies, 424 cases, OR=8, *CI*: 3.83-16.71, I^2^ = 30.1%, *P*<0.05) (**Figure 5**).

#### 3.3.5 Macular central recess thickness

**Figure 6:**
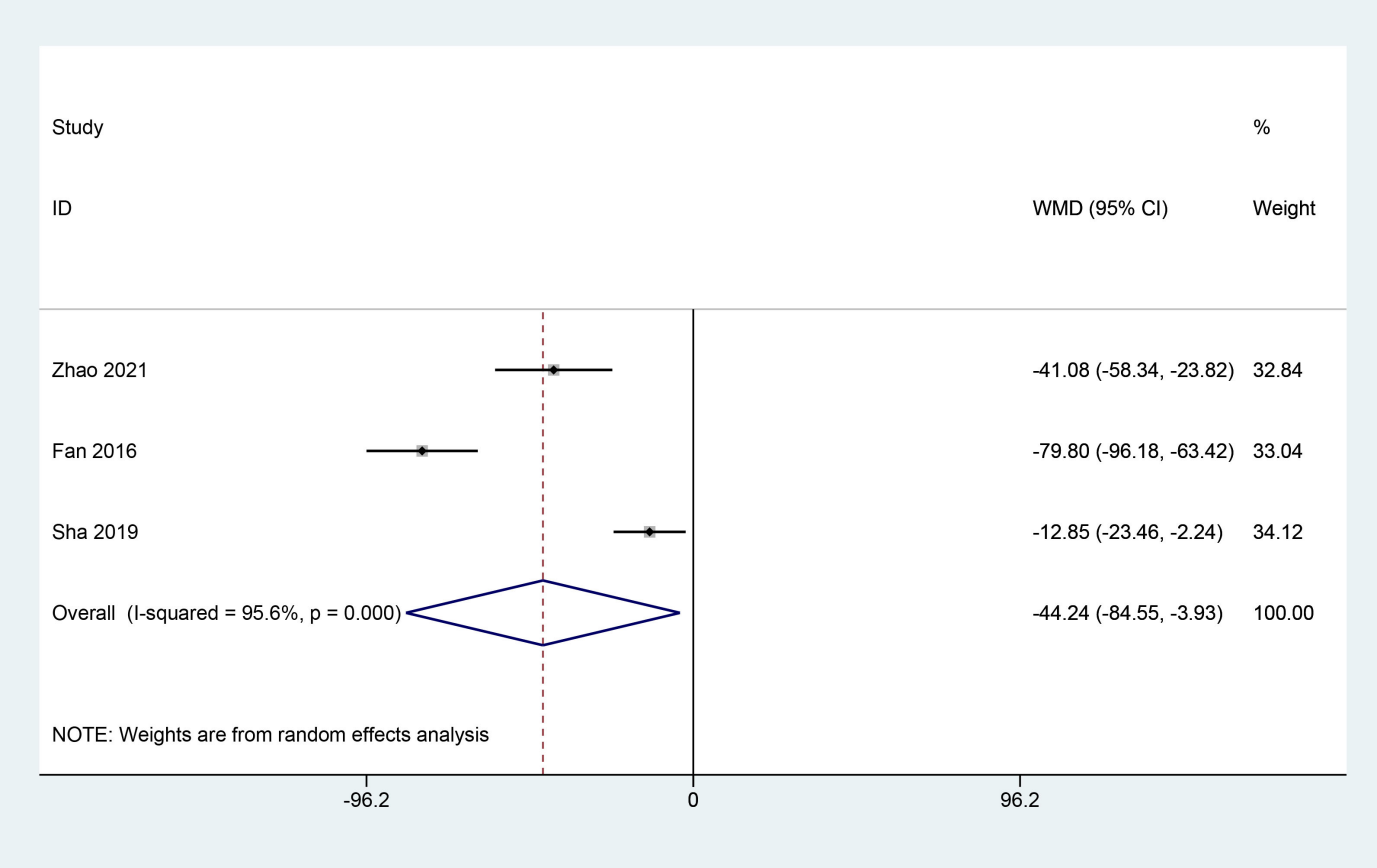
Forest plot of macular central recess thickness.

A total of 3 articles (9, 23, 25) reported on the effect of herbal combination therapy on macular central recess thickness, including 214 patients, and the meta results showed that herbal combination therapy was effective in reducing macular central recess thickness in patients with PDR (3 studies, 214 patients, MD=-44.24, *CI*:-84.55–3.93, *I^2^ = *95.6%, *p*<0.05) (**Figure 6**).

#### 3.3.6 Absorption of vitreous hemorrhage

**Figure 7:**
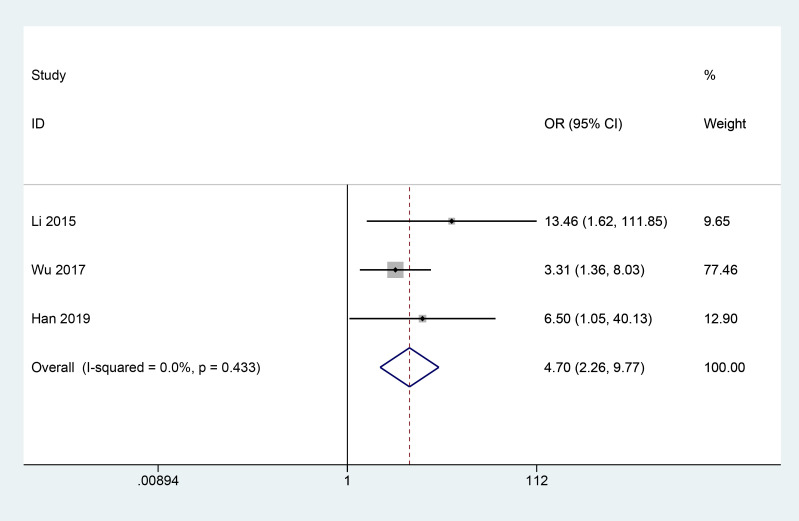
Forest plot of absorption of vitreous hemorrhage.

A total of 3 articles (14, 21, 22) reported the effect of herbal combination therapy on vitreous blood accumulation in patients with PDR. 183 patients were included and the meta results showed that herbal combination therapy was effective in helping vitreous blood accumulation absorption in patients with PDR (3 studies, 183 cases, OR=4.7, *CI*: 2.26-9.77, *I^2^ = *0%, *P*<0.05) (**Figure 7**).

#### 3.3.7 Fasting blood glucose and postprandial 2h blood glucose levels

**Figure 8:**
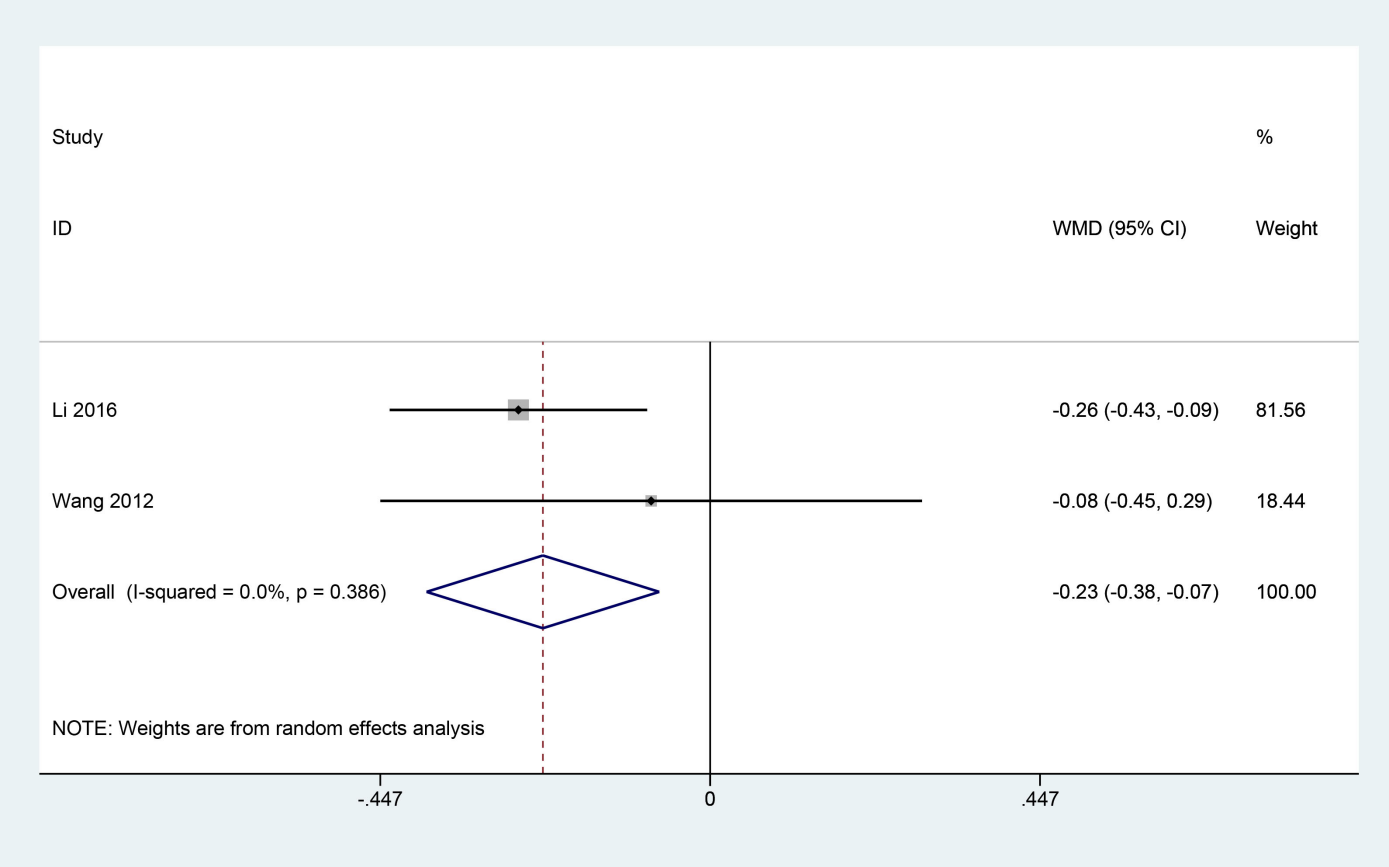
Forest plot of fasting blood glucose levels.

**Figure 9:**
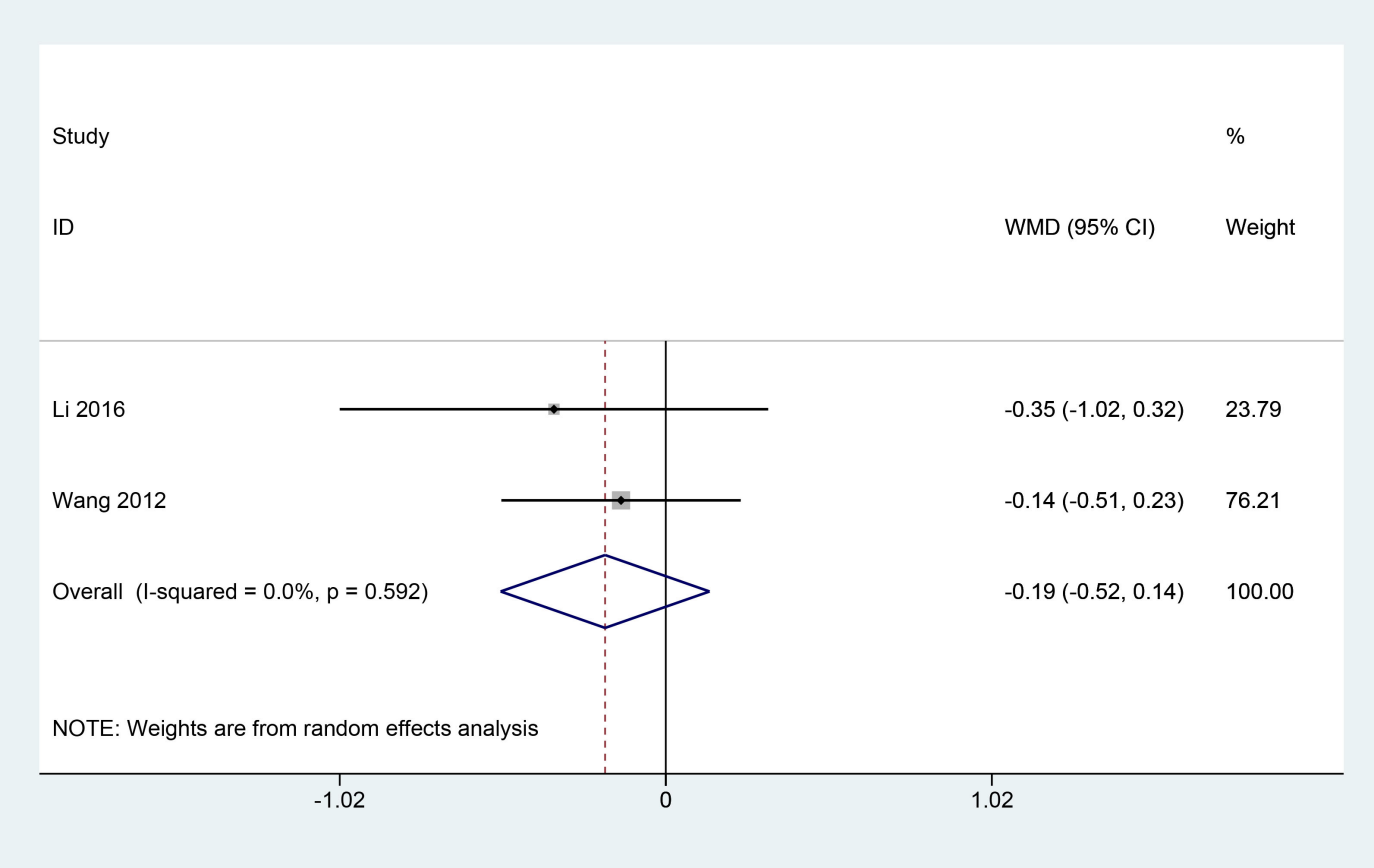
Forest plot of postprandial 2h blood glucose levels.

A total of 2 articles (11, 18) reported the effect of herbal combination therapy on fasting blood glucose and postprandial 2h blood glucose in patients with PDR. 145 patients were included, and the meta results showed that herbal combination therapy was effective in reducing fasting blood glucose in patients with PDR (2 studies, 145 patients, fasting blood glucose: MD=-0.23, *CI*: -0.38-0.07, *I^2^ = *0%. *P*<0.05; postprandial 2h glucose, MD=-0.19, *CI*: -0.52-0.14, *I^2^ = *0%, *P*=0.25) (**Figures 8**, **9**).

### 3.4 Publication bias

**Figure 10:**
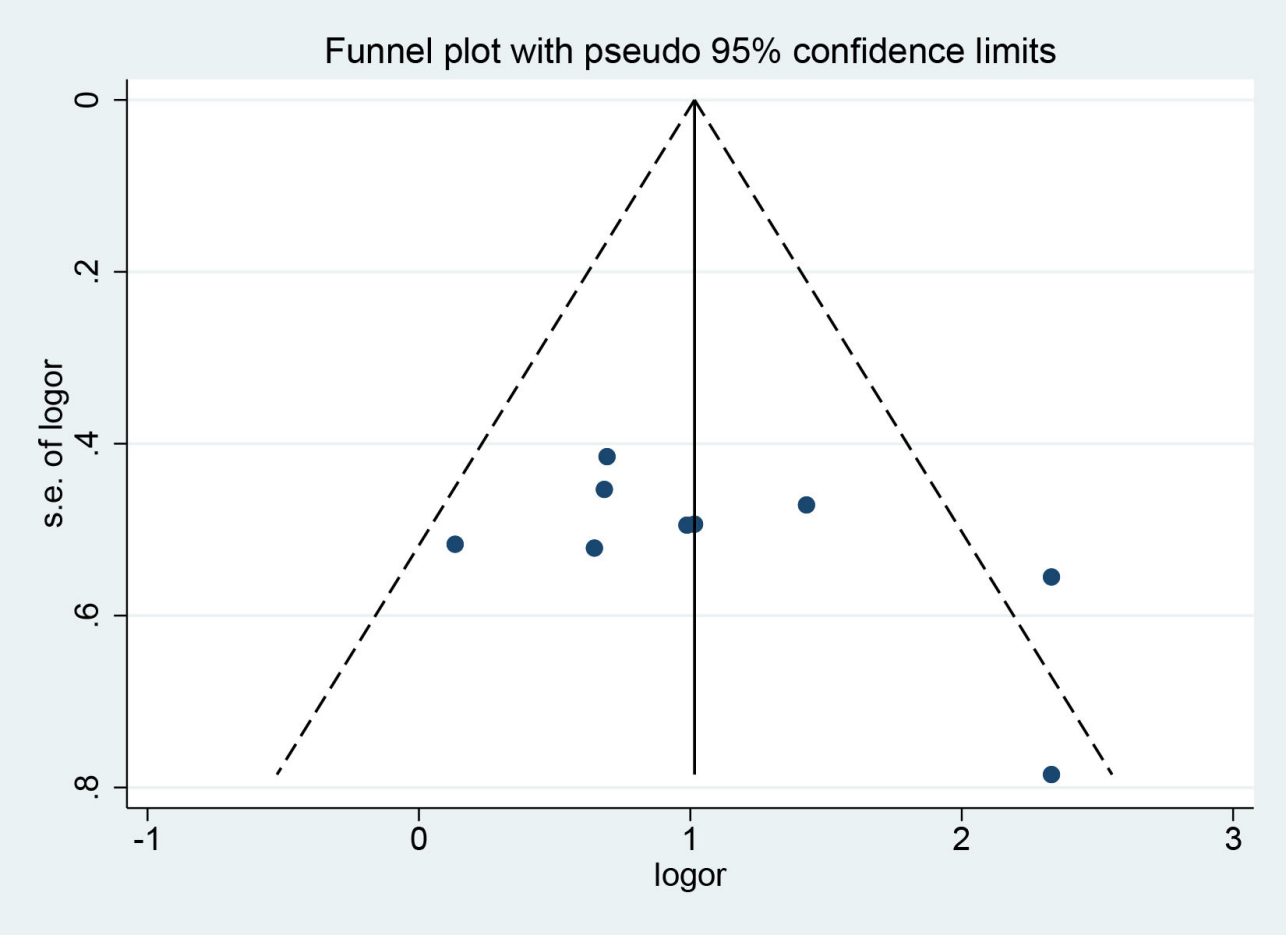
The funnel plot of clinical efficacy.

The clinical efficiency was tested for publication bias. The funnel plot showed that the symmetry was acceptable. Further quantitative analysis was performed using Begg ‘s and Egger ‘s tests. The results showed that there was no publication bias in the outcome index (*P* = 0.251, *P* = 0.112, respectively) (**Figure 10**).

### 3.5 Inclusion of Chinese herbal medicines

In all 18 studies, a total of 69 herbal medicines were applied. After counting the frequency of repetition, it was found that the top four herbal medicines in terms of frequency were Panax notoginseng, Rehmanniae Radix, Astragali Radix and Poria, with their efficacy categories of resolving blood stasis and stopping bleeding, clearing heat and cooling the blood, tonifying Qi, promoting water retention and reducing swelling, respectively (**Table 2**).

**Table 2:** Top 10 Chinese Medicines in Frequency.

Herbs	Frequency	Main Efficacy
Panax Notoginseng	12	Blood Hemostasis
Rehmanniae Radix	10	Heat and Cool Blood
Astragali Radix	9	Energen-Invigorating
Poria	9	Anti-Water Swelling
Alismatis Rhizoma	6	Anti-Water Swelling
Typhae Pollen	6	Blood Hemostasis
Lycii Fructus	6	Invigorating Yin
Salviae Miltiorrhizae Radix Et Rhizoma	6	Activating Blood
Chrysanthemi Flos	5	Diverging Wind Heat
Angelicae Sinensis Radix	5	Hematic

### 3.6 Analysis of the odour and meridian orientation of the included herbs

**Figure 11:**
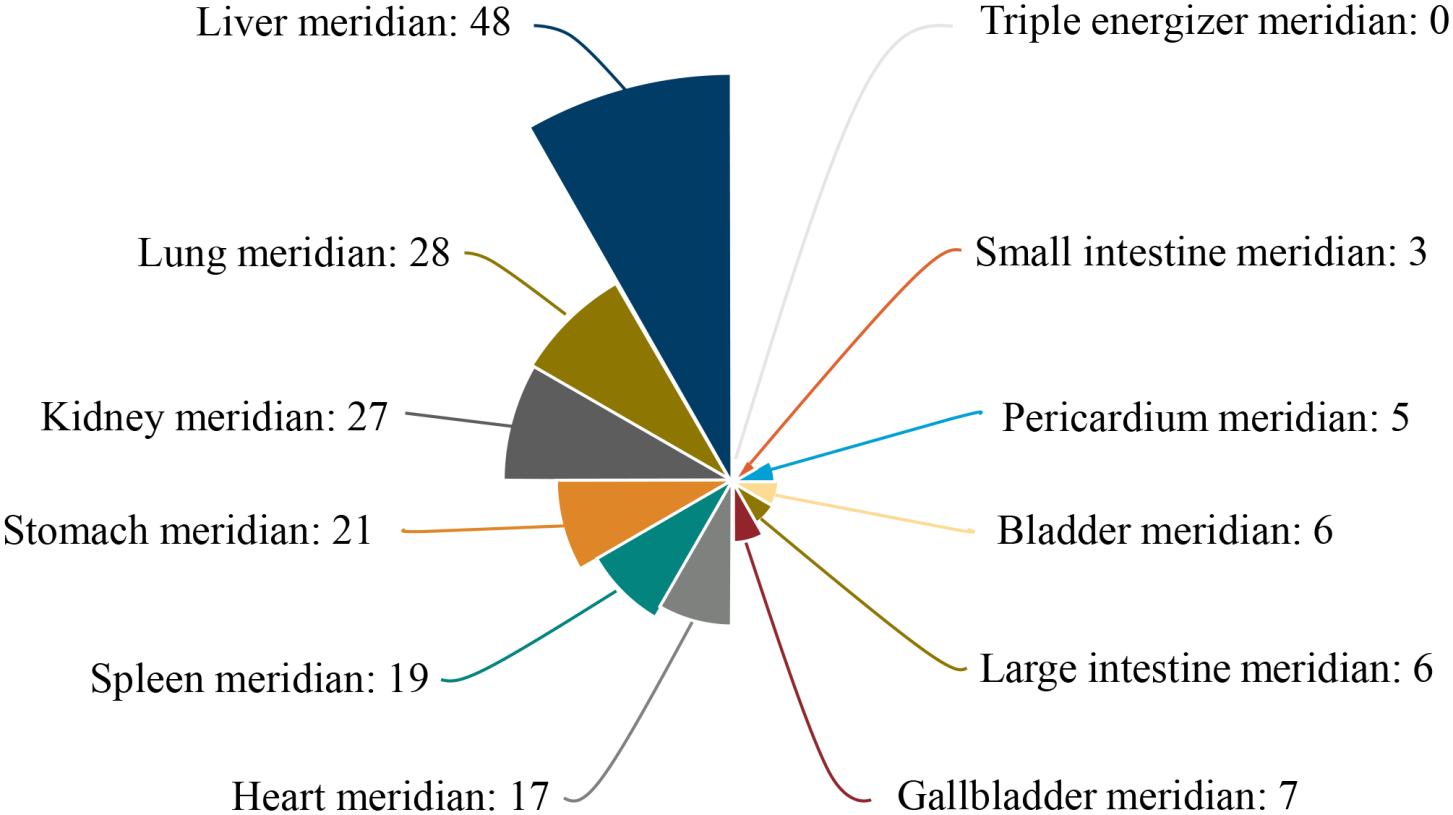
Rose diagram of herbal meridian analysis.

By analysing the nature, taste and meridian orientation information of the 69 herbs intervening in the PDR, the results showed that sweet and bitter herbs accounted for the highest frequency, with the two together accounting for 68.47%; most herbs were on the cold side. In terms of meridian orientation, herbs belonging to the liver meridian accounted for the highest proportion of the 69 species, at 69.57%, which may be related to the theory that the liver opening into the eye in traditional Chinese medicine thinking (**Table 3**) (**Figure 11**).

**Table 3:** Odor and Taste Analysis.

Taste	Frequency	Proportion (%)	Nature	Frequency	Proportion (%)
Sweet	39	35.14	Slight cold	17	26.15
Bitter	37	33.33	Cold	16	24.62
Acrid	17	15.32	Balanced	13	20
Salty	10	9.01	Warm	10	15.38
Sour	4	3.6	Cool	5	7.69
Light	2	1.8	Slight warm	4	6.15
Astringent	2	1.8	Hot	0	0

## 4 Discussion

### 4.1 Summary of main results

Our study included a total of 18 research articles, involving 1,392 patients with PDR. The results show that TCM therapy as an adjunctive treatment effectively improves the clinical outcome and visual acuity of PDR patients and the extent of their fundus lesions. It also has multiple therapeutic effects, such as lowering blood glucose, promoting capillary regression, and accelerating absorption of vitreous hemorrhage, which has multiple positive clinical implications for PDR. The results of publication bias test showed that there was no publication bias in the main indicator, which proved that the results were reliable.

### 4.2 Advantages and limitations of research

The main advantage of this meta-analysis is that it is the first systematic evaluation of the effectiveness of traditional Chinese medicine (TCM) in treating PDR, which preliminary answer the controversial issue of whether TCM is appropriate for PDR treatment. This is of great significance for guiding clinical PDR treatment. Throughout the study, we strictly followed the systematic review method, included almost all relevant and standard RCT studies available online, and the conclusions drawn were comprehensive and robust. Additionally, we conducted bias tests to confirm the reliability of our findings.

However, our study also has some limitations. Firstly, only one of the included studies mentioned the absence of complications and adverse reactions, while the remaining studies did not explore the safety of Chinese herbal medicine in treating PDR. Moreover, most of the studies did not use allocation concealment and blinding methods, nor did they design a placebo group, which may lead to bias in the study results. Finally, as all the literature included in our study came from China, the results we obtained may be more applicable to Chinese PDR patients, and whether TCM is effective for PDR patients in other countries still requires further research.

### 4.3 Analysis of Chinese herbal medicine

The study counted the Chinese medicines used in each study, and the results showed that the top four in terms of replication rate were Panax notoginseng, Rehmanniae Radix, Astragali Radix, and Poria. Panax notoginseng, the root and rhizome of Panax notoginseng, is usually used as a powder in amounts of 1.5-6 g. It is considered by Chinese medicine practitioners to be a critical medicine in the treatment of blood disorders, with the characteristics of “stopping bleeding and activating blood, stopping bleeding without leaving stasis.” Studies have shown that Panax notoginseng can effectively increase the concentration of thrombin and the number of platelets to shorten the clotting time and play a hemostatic role (26). Panax notoginseng contains various types of compounds such as cyclic terpenes and their glycosides, phenolic compounds, sugars, amino acids, and organic acids. These drugs have a variety of pharmacological effects including anti-ageing, hypoglycaemic, anti-inflammatory and immune system boosting (27).

There are relatively few studies on treating DR and PDR with Rehmanniae Radix. However, interestingly, a study from China showed that when the cases of effective treatment of diabetic retinopathy with Chinese herbs in the Chinese Journal Full Text Database (CNKI) were aggregated, Rehmanniae Radix ranked first in the frequency of administration, with 48 times (28), suggesting that Rehmanniae Radix has excellent potential for the treatment of DR and PDR. Its specific mechanisms need to be further explored. Astragalus is the dried root of Astragalus mongolica or Astragalus membranaceus, a leguminous plant, first published in Shen nong Ben Cao Jing and used medicinally in China for more than 2,000 years. It is a sacred tonic for the qi and is one of the most widely used Chinese herbs in China today. Studies have shown that the main anti-diabetic chemicals contained in Astragalus are astragalus polysaccharides, astragalosides, and astragalus flavonoids (29). These drug components can alleviate structural damage and apoptosis of retinal cells caused by the high glucose environment through multiple mechanisms such as hypoglycaemia, anti-oxidative stress, increased free radical scavenging activity, anti-inflammatory response, and inhibition of endoplasmic reticulum stress (30–32). Poria cocos, the dried nucleus of a porous fungus, is a particular herbal medicine that can be used for both medicinal and food purposes. In ancient times, Chinese medicine practitioners believed that regular Poria cocos could strengthen the spleen, promote hydration, tonify the qi and calm the mind, and prolong life, placing it alongside nine other drugs such as ginseng and Ganoderma lucidum as “immortal herbs”. However, there are relatively few records and studies on the use of Poria as a treatment for DR. It ranks highly among the therapeutic drugs for PDR due to its unique effect of promoting the body’s water metabolism (33). In addition to retinal microaneurysms, haemorrhages, and neovascularization, DR often results in diabetic macular oedema (DME) due to the disruption of the blood-retinal barrier and altered hemodynamics, causing extracellular fluid accumulation (34). PDR patients whose disease has progressed to the late stages of DR are usually associated with severe DME with exudative symptoms. Unfortunately, similar to Rehmanniae Radix, there is almost a gap in research exploring Poria alone for treating DME. However, a computer search of the Chinese Journal Full Text Database (CNKI) and Wanfang database for clinical cases related to the treatment of macular oedema ranked Poria first with a frequency of 60 recurrences (35), suggesting that TCM community generally recognizes the efficacy of Poria in the treatment of DME. At the same time, its specific mechanism of action needs to be further explored.

## 5 Conclusion

In summary, our study preliminarily proved that Chinese herbal medicine can effectively improve the clinical outcomes, visual acuity, and degree of retinopathy in patients with proliferative diabetic retinopathy (PDR), as well as reducing blood glucose levels, aiding capillary regression, and alleviating vitreous hemorrhage. These conclusions have significant implications for resolving controversies surrounding the application of traditional Chinese medicine (TCM) in the field of PDR. In addition, data mining of herbal medicines included in the literature reveals that researchers prefer to use herbs with sweet or bitter taste and slight cold or cold properties that belong to the liver meridian to treat PDR. In future studies, we need a larger sample of high-quality, multi-centre RCT studies to provide evidence for treating PDR with Chinese herbs. These studies should also focus on exploring safety.

## Author contributions

This review was created by BGH, BSH, ZS, MS, DL, TX, CL and YC. The First drafts of the article were written by BGH and BSH, and further revisions were contributed to by the other co-authors. The final version of the work was approved by all authors. DL obtained funding for this study. All authors contributed to the article and approved the submitted version.
